# Evaluation of Parkin in the Regulation of Myocardial Mitochondria-Associated Membranes and Cardiomyopathy During Endotoxemia

**DOI:** 10.3389/fcell.2022.796061

**Published:** 2022-02-21

**Authors:** Matthew Kim, Azadeh Nikouee, Yuxiao Sun, Qing-Jun Zhang, Zhi-Ping Liu, Qun Sophia Zang

**Affiliations:** ^1^ Department of Surgery, Burn & Shock Trauma Research Institute, Loyola University Chicago Stritch School of Medicine, Maywood, IL, United States; ^2^ Department of Surgery, University of Texas Southwestern Medical Center, Dallas, TX, United States; ^3^ Internal Medicine-Cardiology Division, University of Texas Southwestern Medical Center, Dallas, TX, United States

**Keywords:** mitophagy, mitochondria, parkin, cardiac dysfunction, inflammation, sepsis, endotoxemia

## Abstract

**Background:** Mitochondrial deficiency is a known pathology in sepsis-induced organ failure. We previously found that mitochondria-associated membranes (MAMs), a subcellular domain supporting mitochondrial status, are impaired in the heart during endotoxemia, suggesting a mechanism of mitochondrial damage occurred in sepsis. Mitophagy pathway *via* E3 ubiquitin ligase Parkin and PTEN-induced kinase 1 (PINK1) controls mitochondrial quality. Studies described here examined the impact of Parkin on cardiac MAMs and endotoxemia-induced cardiomyopathy. Additionally, point mutation W403A in Parkin was previously identified as a constitutively active mutation *in vitro*. *In vivo* effects of forced expression of this mutation were evaluated in the endotoxemia model.

**Methods:** Mice of wild type (WT), Parkin-deficiency (*Park2* ^*−/−*^), and knock-in expression of Parkin W402A (human Parkin W403A) were given lipopolysaccharide (LPS) challenge. Cardiac function was evaluated by echocardiography. In the harvested heart tissue, MAM fractions were isolated by ultracentrifugation, and their amount and function were quantified. Ultrastructure of MAMs and mitochondria was examined by electron microscopy. Mitochondrial respiratory activities were measured by enzyme assays. Myocardial inflammation was estimated by levels of pro-inflammatory cytokine IL-6. Myocardial mitophagy was assessed by levels of mitophagy factors associated with mitochondria and degrees of mitochondria-lysosome co-localization. Parkin activation, signified by phosphorylation on serine 65 of Parkin, was also evaluated.

**Results:** Compared with WT, *Park2* ^*−/−*^ mice showed more severely impaired cardiac MAMs during endotoxemia, characterized by disrupted structure, reduced quantity, and weakened transporting function. Endotoxemia-induced cardiomyopathy was intensified in *Park2* ^*−/−*^ mice, shown by worsened cardiac contractility and higher production of IL-6. Mitochondria from the *Park2* ^*−/−*^ hearts were more deteriorated, indicated by losses in both structural integrity and respiration function. Unexpectedly, mice carrying Parkin W402A showed similar levels of cardiomyopathy and mitochondrial damage when compared with their WT counterparts. Further, Parkin W402A mutation neither enhanced mitophagy nor increased Parkin activation in myocardium under the challenge of endotoxemia.

**Conclusion:** our results suggest that Parkin/PINK1 mitophagy participates in the regulation of cardiac MAMs during endotoxemia. Point mutation W402A (human W403A) in Parkin is not sufficient to alleviate cardiomyopathy induced by endotoxemia *in vivo*.

## Introduction

Sepsis is currently a leading cause of fatality in critical care units (Singer et al., 2016). Despite improvements in antibiotic therapies and critical care techniques (Levy et al., 2010), reported incidences of sepsis are still rising (Iwashyna et al., 2010). Thus, to understand the molecular mechanisms of sepsis pathogenesis and to explore new therapeutic interventions for this critical condition are in urgent need.

Cardiomyopathy is a critical component of multi-organ failure occurring in severe sepsis, and it often serves as a main predictor of poor outcomes (Blanco et al., 2008; Zanotti-Cavazzoni and Hollenberg, 2009; Walley, 2018). Previously, research from others and our laboratory demonstrated that impairments in mitochondria is a major drive inducing cardiac failure in sepsis, likely due to deficiency in energy production, elevation in oxidative stress, and overproduction of mitochondria-derived danger-associated molecular patterns (DAMPs) (Zang et al., 2007; Zang et al., 2012a; Zang et al., 2012b; Yao et al., 2015). The quality and quantity of mitochondria are tightly regulated by a multi-step process that includes mitochondrial biogenesis, mitochondrial fusion-fission dynamics, and mitophagy-mediated recycling. Interestingly, studies in recent years revealed that mitochondria-associated membranes (MAMs), the regions of close physical connection between mitochondrial outer membrane and membranes of endoplasmic reticulum (ER), are an important subcellular domain providing additional control to ensure mitochondrial properties (Mannella et al., 1998; Axe et al., 2008; Hayashi-Nishino et al., 2009; Patergnani et al., 2011; Raturi and Simmen, 2013; Giorgi et al., 2015). Proper mitochondrial physiology is dependent on the specific communication between mitochondria and ER for transporting Ca^2+^ (Patergnani et al., 2011) and lipids (Vance, 2014). Further, MAMs also function as a signaling hub, filled with dynamically translocated molecules that are involved in important cellular events of protein sorting, ER stress, apoptosis, inflammation, and autophagy (Poston et al., 2013; van Vliet et al., 2014). Currently, abnormalities in MAMs have been detected in disease models that involve mitochondrial dysfunction as a major pathogenesis component, such as in neurodegenerative diseases, diabetes, obesity, and infectious diseases (Bach et al., 2003; Ishikawa et al., 2009; Zorzano et al., 2009; Area-Gomez et al., 2012; De Strooper and Scorrano, 2012; Park et al., 2013). However, whether MAMs play a significant role in sepsis-induced cardiomyopathy has not been well understood. We recently found that endotoxemia caused MAM impairments in the heart tissue, suggesting a signaling mechanism underlying the deficiencies of mitochondria in septic hearts (Sun et al., 2021). Future investigations to address how aberration in MAMs occurs and its related pathological consequences during sepsis are important to identify novel therapeutic targets.

We previously found that the promoting autophagy *via* autophagy initiation factor Beclin-1 limited inflammation, governed mitochondrial quality control, reduced mitochondria-derived DAMPs, and thus, improved cardiac function during endotoxemia (Sun et al., 2018a). We also obtained results suggesting that Beclin-1 removed dysfunctional mitochondria by selectively activating an adaptive mitophagy *via* PTEN-induced kinase 1 (PINK1) and E3 ubiquitin ligase Parkin. Our finding is also in consistency with previous reports implicating the adaptive feature of PINK1-Parkin mitophagy in cardiac performance (Kubli et al., 2013; Piquereau et al., 2013). While disrupted Parkin expression was shown to cause insufficient mitophagy, resulting in an accumulation of damaged mitochondria and eventually caused cardiac failure (Kubli et al., 2013; Piquereau et al., 2013; Dorn, 2016), activation of Parkin was expected to provide, at least partial, cardiac protection. Recent studies using *in vitro* structure-guided mutagenesis and evaluation in cultured cells successfully selected several activating mutant forms of Parkin, within which mutant W403A held the highest promise of developing potential Parkin-targeted therapeutic strategies (Tang et al., 2017; Yi et al., 2019). In the investigation presented in this report, by using genetically engineered mouse strains with knockout expression of Parkin and knock-in expression of Parkin W403A, we evaluated the functional significance of Parkin in the regulation of myocardial MAMs, mitochondria, and cardiomyopathy in the model of endotoxemia. We also examined whether introducing mutant W403A Parkin may deliver any beneficial cardiac protection during endotoxemia*.*

## Materials and Methods

### Experimental Animals

**FIGURE 1:**
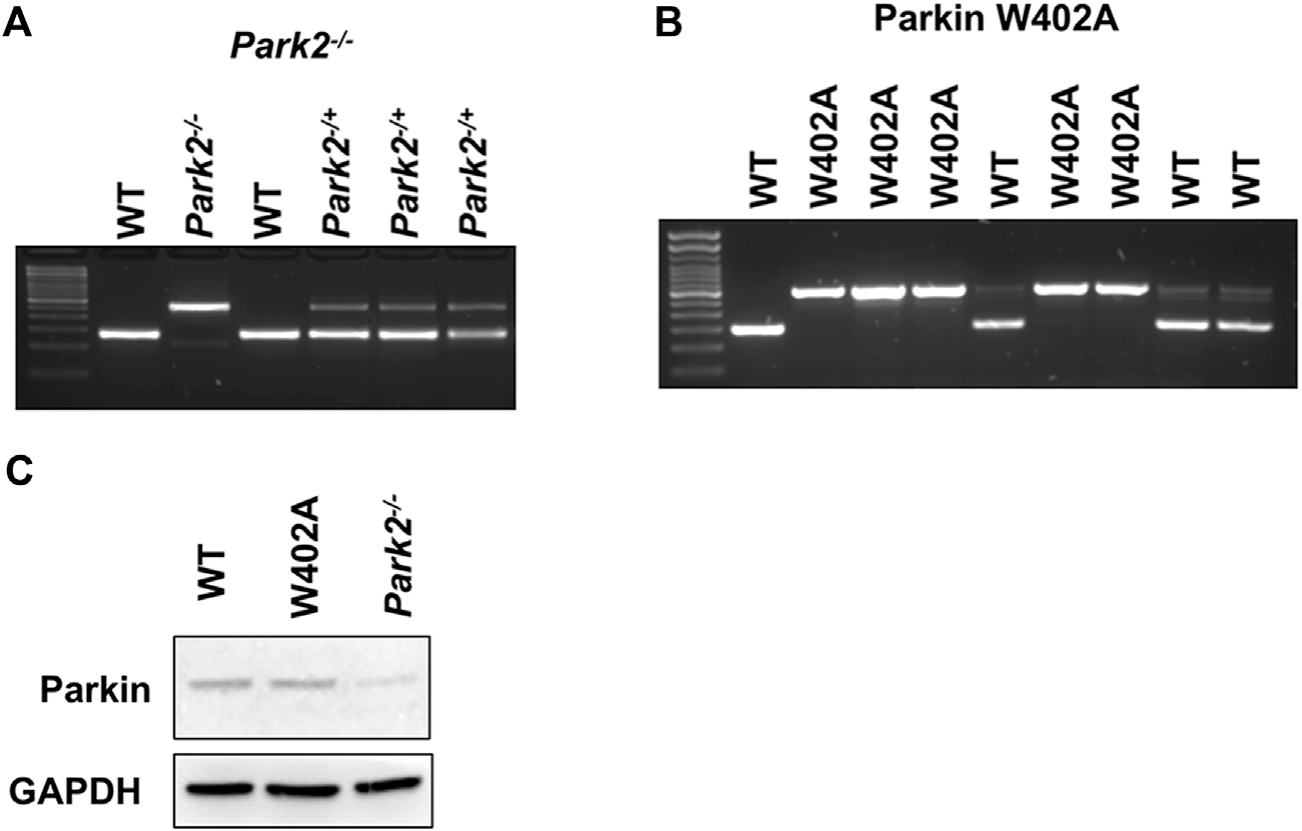
Genotyping and the expression of Parkin in WT, *Park2* ^*−/−*^, and Parkin W402A mice. Pups of strain *Park2* ^*−/−*^ **(A)** and Parkin W402A **(B)** breeders were subjected to genotyping PRC according to genotyping protocols provided by the vendor. Protein expression of Parkin was verified in the heart tissue of adult WT, *Park2* ^*−/−*^, and Parkin W402A mice by western blot **(C).** Images shown are representative of at least 5 independent experiments.

Mouse strains that carry Parkin knockout (*Park2* ^*−/−*^) and knock-in of Parkin W402A mutation (Parkin W402A KI) (W403A in human Parkin) were obtained from the Jackson Laboratory (Bar Harbor, ME) (stock numbers 006582 and 029317). According to the vender’s suggestion, wild type mice C57BL/6NJ (Jackson Laboratory, stock number 005304) were chosen as the appropriate control. All animals were conditioned in-house for 5–6 days after arrival with commercial diet and tap water available at will. To properly maintain the colonies, genotyping was performed in each individual animal according to the vender’s protocols (protocol 22730 for *Park2* ^*−/−*^ and protocol 20141 for Parkin W402A KI). Figure 1 provided example results of genotyping verification for the colony management

The care of animal work described in this study was reviewed by and conducted under the oversight of UTSW institutional animal care and use committee and conformed to the National Research Council’s “Guide for the Care and Use of Laboratory Animals” when establishing animal research standards.

### Endotoxemia Model

Male mice, 8–12 weeks old, were weighed to determine the amount of lipopolysaccharide (LPS) (MilliporeSigma, Burlington, MA; catalog number L3012) required to achieve indicated doses and administered intraperitoneally (i.p.) in a volume of 100 μl per mouse. Sterile endotoxin-free PBS was used as a vehicle control in sham groups.

### Echocardiography

Transthoracic echocardiograms were recorded in sedated mice using Visualsonics Vevo 2,100 small animal echocardiography machine. Views were taken in planes that approximated the parasternal short-axis view and the apical long-axis view in humans.

### Transmission Electron Microscopy

Hearts were retrograde perfused (buffer: 4% paraformaldehyde/1% glutaraldehyde/0.1 M Na Cacodylate, pH7.4). Small blocks of tissue from the midsection of the left ventricular wall were fixed (buffer: 2.5% glutaraldehyde/0.1 M Na Cacodylate, pH7.4). Sections (75–80 nm) were cut using a Leica Ultramicrotome and examined under TEM. Quantification of MAM areas was achieved by Image J software.

### Preparation of Serum and Tissue Lysates

Freshly collected blood was immediately centrifuged at 3,000 g for 15 min at 4°C to isolate serum. The serum preparations were then allocated and stored at −80°C until analyzed. Tissues were harvested, washed in PBS, snap clamp frozen, and kept at −80°C. Tissue lysates were prepared using tissue protein extraction reagent (Thermo Fisher Scientific, Rockford, IL; catalog number 78510). Protein concentrations were quantified using detergent compatible Bradford assay kit (Thermo Fisher Scientific; catalog number 23246).

### Preparation of MAM and Mitochondria Fractions

Heart tissues were harvested, washed in PBS, snap-clamp frozen, and kept at −80°C until used. Procedures for the isolation of MAMs and mitochondria were performed according to a previously established procedure (Sun et al., 2021). Briefly, tissue pieces of one mouse heart were homogenized in 1 ml IB_heart_ buffer (220 mM mannitol, 70 mM sucrose, 10 mM HEPES, and 1 mM EGTA, pH7.4) using a Potter-Elvehjem PTFE pestle and glass homogenizer (MilliporeSigma; catalog number P7734), which was driven by a stirrer motor with electronic speed controller (Cole-Palmer, Vermon Hills; catalog number EW-04369-10) by 40 strokes at a speed of 1,500 rpm followed by another 40 strokes at 800 rpm. Crude mitochondrial fractions were then obtained by differential centrifugation in the following two steps. First, the homogenized heart lysates were subjected to twice-repeated centrifugation at 740 g for 5 min to remove unbroken cells and nuclei. Second, the supernatant mixtures were centrifuged at 9,000 g for 10 min to collect pellets. These pellets were then resuspended in freshly prepared mitochondria-resuspension buffer (MRB; 250 mM mannitol, 5 mM HEPES, and 0.5 mM EGTA, pH7.4) and subjected to twice-repeated centrifugation at 10,000 g for 10 min to collect crude mitochondria. The crude mitochondria pellets were then resuspended in MRB at the ratio of 0.5 ml MRB per heart and subjected to ultracentrifugation (Sorvall MX 120 Plus Micro-Ultracentrifuge with rotor S50-ST; Thermo Scientific; catalog number 50135645) to isolate MAMs and pure mitochondria (PM) by the following three steps. First, in each 7 ml ultracentrifuge tube, 6 ml of freshly made percoll medium [225 mM mannitol, 25 mM HEPES (pH7.4), 1 mM EGTA, and 30% percoll (v/v)] was layered with 0.5 ml of crude mitochondria resuspension and 0.5 ml of MRB, from the bottom to the top, and centrifuged at 95,000 g for 30 min. Fractions of mitochondria, dense bands located approximately at the bottom, and MAMs, diffused white bands located above the mitochondria, were collected. Second, the collected bands of mitochondria and MAMs were diluted 10 times with MRB and further centrifuged at 6,300 g for 10 min. Third, mixtures of MAMs bands and mitochondria bands were centrifuged at 100,000 g for 1 h. For fractions of MAMs, the pellets were collected and stored at −80°C until used. For fractions of mitochondria, the pellets were collected and resuspended with MRB again, followed by another two washes by centrifugation at 6,300 g for 10 min, and the PM pellets were then collected and stored at -80°C until used. All chemicals were purchased from MilliporeSigma.

### Quantification of Mitochondrial Phospholipids

Levels of phospholipids in mitochondria were measured with a phospholipid assay kit (MilliporeSigma; catalog number MAK122) as described previously (Sun et al., 2021). Briefly, fractions of mitochondria were diluted to 1–2.5 μg protein per assay using the assay buffer provided. Each reaction mix was set up by adding a prepared sample or standard to phospholipid D that degrades phospholipids to release choline. The amount of choline was determined with choline oxidase and an H_2_O_2_ specific dye. A colorimetric reading at wavelength 570 nm was proportional to the phospholipid concentration in the sample. Results were calculated according to the standard curve and normalized by protein amount per sample, and measurements were performed in triplicates.

### Mitochondrial Respiratory Complex I, II, and III Enzyme Assays

The activities of mitochondrial complexes were measured using enzyme assay kits according to manufacturer’s protocols (Abcam, Cambridge, MA; catalog numbers ab109721 and ab109905). Freshly isolated mitochondrial pellets were resuspended in PBS supplemented with 10% detergent provided in the kits. Protein concentrations of these mitochondrial lysates were estimated and 25 μg (for complex I) or 100 μg (for complex II + III) mitochondrial protein was used per reaction. Enzyme activities were measured spectrophotometrically in triplicate and expressed as changes of absorbance per minute per mg of protein.

### Measurements of Cytokines by Enzyme-Linked Immunosorbent Assay

Cytokine levels in serum or in total tissue lysates were measured using Bio-Plex Mouse Cytokine Panel A 6-Plex (Bio-Rad, Hercules, CA; catalog number M6000007NY) according to vendor’s instructions. Results were normalized by volume of serum samples or by protein amount in tissue lysates.

### Western blots

Prepared SDS-PAGE protein samples were loaded and run on 20–4% tris-glycine stain free ready gels (Bio-Rad) and transferred to PVDF membranes. Stain free total protein measurement Membranes were blocked with 5% nonfat milk-PBS at room temperature for 1 h and subsequently probed with antibodies against Parkin (Santa Cruz Biotechnology, Santa Cruz, CA, catalog number sc-30130), phospho-Parkin (Ser65), LC3II (Cell Signaling, Danvers, MA; catalog number 368665 and 4,108), cytochrome C, FALC4, VDAC1, and PEN2 (Abcam, Cambridge, MA; catalog numbers ab110325, ab155282, ab14734, and ab18189), PINK1 (Novus Biologicals, Littleton, CO, catalog number BC100-494), and GAPDH (Millipore, Billerica, MA, catalog number MAB374). The membranes were then rinsed and incubated with corresponding horseradish peroxidase-conjugated secondary antibody (Rockland Immunochemicals, Pottstown, PA; catalog numbers 170-6515 and 170-6516). Antibody dilutions and incubation time were according to manufacturer’s instructions. At the end, membranes were rinsed, and bound antibodies were detected by using SuperSignal West Pico Chemiluminescent Substrate (Thermo Scientific; catalog number 34077). Densitometry analysis was performed using Bio-Rad ChemiDoc MP Imaging System.

### Immunostaining

Fresh heart tissues were fixed in 4% paraformaldehyde, transferred to 18–10% sucrose in PBS, and embedded in OCT. Samples were sectioned at 8 μm, air-dried and stored at −80°C until used. Frozen slides were then thawed, permeabilized, blocked with 3% donkey serum (Jackson ImmunoResearch catalog number 017-000-121) in PBS, and subjected to staining by a rat monoclonal anti-Lamp1 (Santa Cruz Biotechnology, Santa Cruz, CA, catalog number sc-1992; 1:300) or a rabbit monoclonal anti-Mfn2 (Cell Signaling, Danvers, MA, catalog number 9482; 1:100 at room temperature, 1 h). Upon completion of secondary antibody incubation with Alexa Fluor 448 conjugated donkey anti-rat IgG (Thermo Scientific; catalog number A-21208; 1:300) or Alexa Fluor 647 conjugated donkey anti-rabbit IgG (Thermo Scientific; catalog number A-31573; 1:300), the slides were washed, sealed with DAPI/antifade mounting solution (Thermo Fisher Scientific, Rockford, IL; catalog number 36931), and examined. Images were acquired with a LSM 510 confocal microscope equipped with an Axio Observer Z1 motorized inverted microscope and Zen software (Carl Zeiss Microscopy) and analyzed offline with Imaris software (version 9.5, Bitplane).

### Statistical Analysis

All data were expressed as mean ± SEM of at least 3 independent experiments using 4-6 animals/group. Data were analyzed by GraphPad using two-way ANOVA test for comparisons of multiple groups. Differences were considered statistically significant as *p* ≤ 0.05.

## Results

### Cardiomyopathy in Mouse Strains of WT, *Park2* ^*−/−*^, and Parkin W402A During Endotoxemia

Previous studies in animal models with Parkin deficiency showed that the Parkin-mediated mitophagy is essential for cardiac performance and mitochondrial physiology in myocardium (Kubli et al., 2013; Piquereau et al., 2013; Sun et al., 2018a). On the other hand, *in vitro* analysis revealed that point mutation W403A in Parkin resulted in a constitutive activation, and this mutation was shown to enhance Parkin activity and promote PINK1-Parkin mitophagy (Tang et al., 2017; Yi et al., 2019).

Therefore, we speculated that a knock-in expression of this active mutant Parkin might provide protection in cardiac disease models *in vivo.* We previously established a mouse model of survival endotoxemia, in which cardiomyopathy is induced by a toxic dose of LPS (Sun et al., 2018a; Sun et al., 2018b). In this report, experiments were designed to evaluate the effects of disrupted *parkin* expression and knock-in, forced expression of Parkin mutant W402A (human W403A) on cardiomyopathy during endotoxemia. Mice strains carrying *parkin* knockout, *Park2* ^*−/−*^, and Parkin W402A are commercially available. These mice are viable with no apparent abnormalities at baseline when observed up to 12 months (Simpkins et al., 2005; Kubli et al., 2013). Proper genotyping of the mouse strains was performed according to the vendor’s protocols, as described in the section of *Materials and Methods* (Figure 1).

**FIGURE 2:**
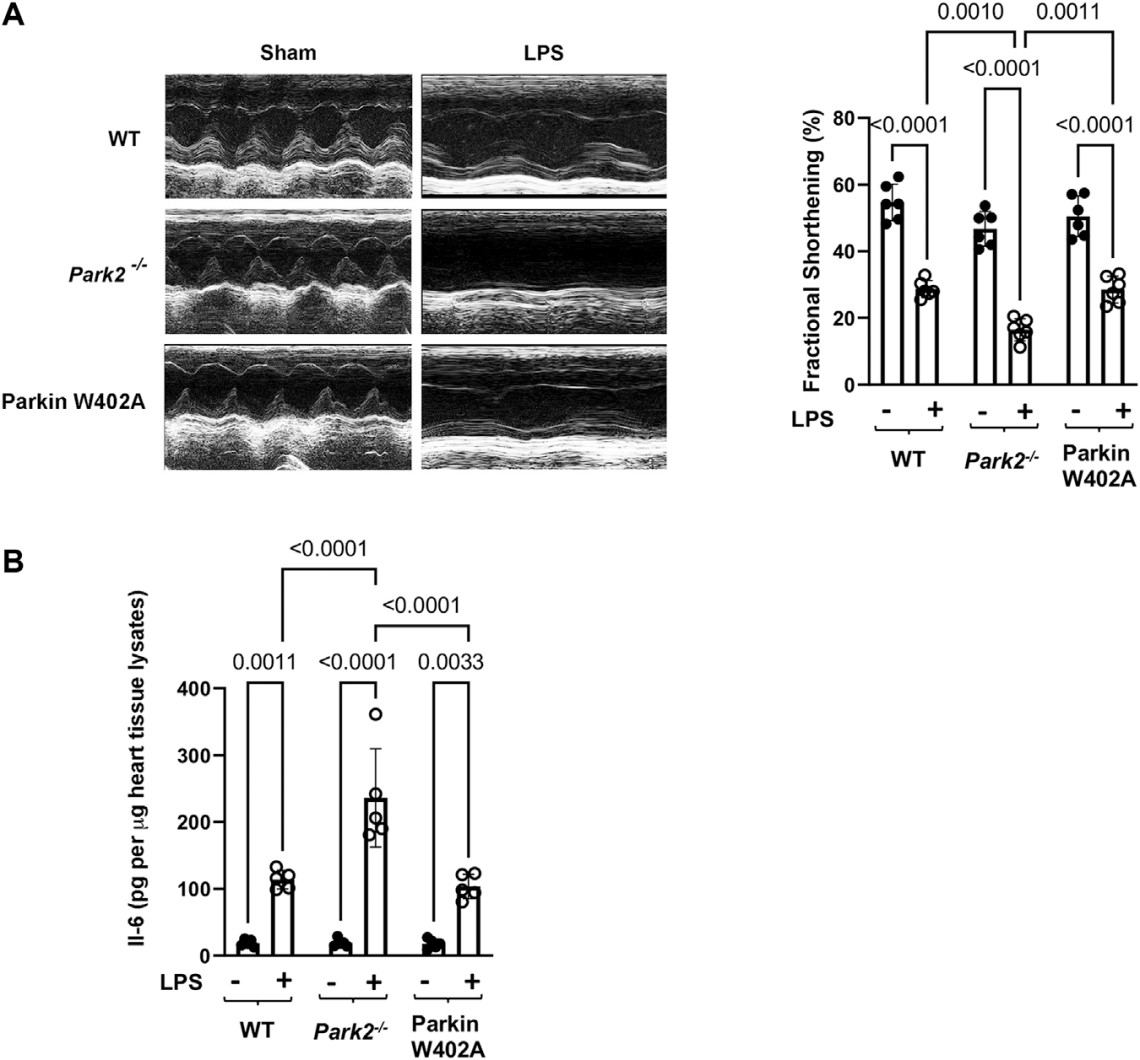
Cardiac outcomes in LPS challenged WT, *Park2* ^*−/−*^, and Parkin W402A mice. Mice were given 5 mg/kg LPS or PBS (in shams) *via* i.p... At 18 h post LPS challenge, echocardiography was applied to measure cardiac function **(A).** The heart tissue was then harvested, and IL-6 levels were quantified in the tissue lysates by ELISA **(B).** All values are presented as means ± SEM. *p* values with less than 0.05 showing significant differences, are labeled (*n* = 5).

WT, *Park2* ^*−/−*^, and Parkin W402A mice was given LPS challenge at 5 mg/kg and subjected to the assessment of cardiac performance by echocardiography at 18 h post-challenge. As the results summarized in Figure 2A, we found that LPS caused significant decreases in cardiac contractility of all three strains. The fractional shortening was reduced about 40–50% by LPS in the WT and Parkin W402A strains, whereases LPS induced the worst cardiac performance in *Park2* ^*−/−*^ mice–a more than 60% reduction in contractility. This observation of cardiac dysfunction in *Park2* ^*−/−*^ mice is consistent with the literature (Kubli et al., 2013; Piquereau et al., 2013), likely due to the halted mitophagy that causes overaccumulation of deficient mitochondria in myocardium. However, the result obtained in Parkin W402A mice is unexpected since this mutant was suggested carrying Parkin activation in previous published *in vitro* studies (Tang et al., 2017; Yi et al., 2019).

Overwhelming inflammation is characteristic in sepsis, which is hypothesized as the main reason for causing multi-organ failure. We previously showed that IL-6 is the most responsive cytokine to LPS challenge in the heart tissue (Sun et al., 2018b). To evaluate Parkin in myocardial inflammation, heart tissue from WT, *Park2* ^*−/−*^ *,* and Parkin W402A mice was harvested 18 h post LPS challenge and levels of IL-6 in tissue lysates were compared. Results summarized in Figure 2B showed that LPS triggered an over 5-fold increase in IL-6 production in both WT and Parkin W402A mice, and the most dramatic stimulation of IL-6 by LPS was detected in the *Park2* ^*−/−*^ hearts with a ∼ 12-fold increase. Thus, the data suggest Parkin as a critical control over the stimulation of cardiac inflammation during endotoxemia. However, like its effect on cardiac function, Parkin mutant W402A provided little protection again LPS-induced inflammatory response in the heart tissue.

### Status of Myocardial Mitochondria in Mouse Strains of WT, *Park2* ^*−/−*^, and Parkin W402A During Endotoxemia

**FIGURE 3:**
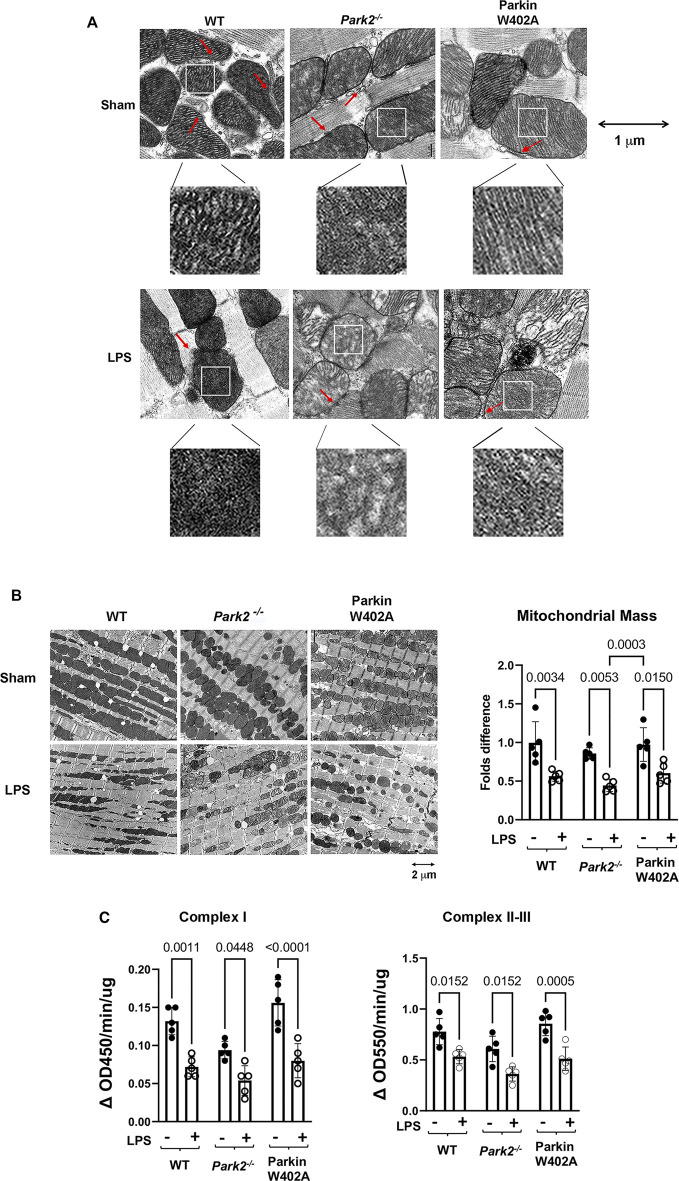
Ultrastructure of cardiac mitochondria in WT and *Park2* ^*−/−*^ mice pre- and post-LPS challenge. Mice were given 5 mg/kg LPS or PBS (in shams) *via* i.p., and heart tissues were harvested 18 h post LPS challenge. Ultrastructure of mitochondria in myocardium was observed under TEM. **(A).** Images show the details of individual mitochondria in myocardium at the indicated magnification. Each white square-circled area was enlarged 9-time for viewing the inside of mitochondria. Red arrows indicate the locations of MAMs. **(B).** Images show the numbers and sizes of mitochondria in myocardium at the indicated magnification. Numbers of intact mitochondria in 10 × 10 μm^2^ were counted, and >5 areas per sample were examined. Results were expressed as folds compared with that in the group of WT shams. In A and B, images are representative of at least 5 animals per group. **(C).** Enzymatic activities of mitochondrial respiratory complexes I and II/III were quantified in the mitochondrial fractions isolated from the heart tissue harvested at 18-h post LPS challenge. All values are presented as means ± SEM. *p* values with less than 0.05, showing significant differences, are labeled (*n* = 5).

Mitochondrial damage is a known mechanism of sepsis pathology, and it is closely associated with sepsis severity. In this study, we compared LPS-induced changes in cardiac mitochondrial structure and function in WT, *Park2* ^*−/−*^, and Parkin W402A mice. Following given LPS challenge at 5 mg/kg, and the left ventricular wall of hearts was harvested at 18 h post challenge and applied to the assessment by transmission electron microscopy (TEM). As shown in Figure 3A, we observed that LPS triggered a significant loss of mitochondrial outer membrane integrity and disruption of inner membrane cristae structures in all three strains. There was no evident difference detected between the WT and Parkin W402A mice. When the heart tissue slides were examined in a larger field under TEM (Figure 3B), numbers of mitochondria in a fixed area of myocardium were counted and statistical analysis was applied. Data indicated that LPS stimulated a trend of reduction in the mass of total mitochondria, which was also associated with an accumulation of small-sized dysfunctional mitochondria in all three mouse strains. Quantification of the intact mitochondria showed that LPS challenge reduced the total number of mitochondria about 50% in WT and Parkin W402A mice and about 60% in *Park2* ^*−/−*^ mice.

To evaluate mitochondrial function, mitochondrial fractions isolated from the heart tissue harvested at the same time point were applied to the measurements of the enzymatic activities of mitochondrial respiratory complexes I and II/III. Summarized in Figure 3C, data indicate that LPs induced significant decreases in the enzymatic activities of mitochondrial respiratory complexes I-III in animals of all strains. In addition, this response in Parkin W402A mice was similar to that in the WT. These results showed that LPS stimulates mitochondrial damage, including structural abnormality and functional deficiencies, in the heart of WT, *Park2* ^*−/−*^, and Parkin W402A mice, among which responses in the strain of *Park2* ^*−/−*^ was the most severe. Data also demonstrated that introducing Parkin mutation W402A has little effect on this LPS-induced harmful response.

### Parkin is Essential to Maintain the Structure and Function of Myocardial MAMs During Endotoxemia

MAMs are an essential structural entity to support mitochondrial health (Mannella et al., 1998; Axe et al., 2008; Hayashi-Nishino et al., 2009; Patergnani et al., 2011; Raturi and Simmen, 2013; Giorgi et al., 2015). Recently, our investigation revealed that endotoxemia causes impairments in cardiac MAMs (Sun et al., 2021). Since PINK1-Parkin mitophagy is a major quality control mechanism for mitochondria (Kubli et al., 2013; Piquereau et al., 2013; Sun et al., 2018b), we next examined whether myocardial MAMs are also subjected to regulation *via* the PINK1-Parkin signaling axis during endotoxemia.

**FIGURE 4:**
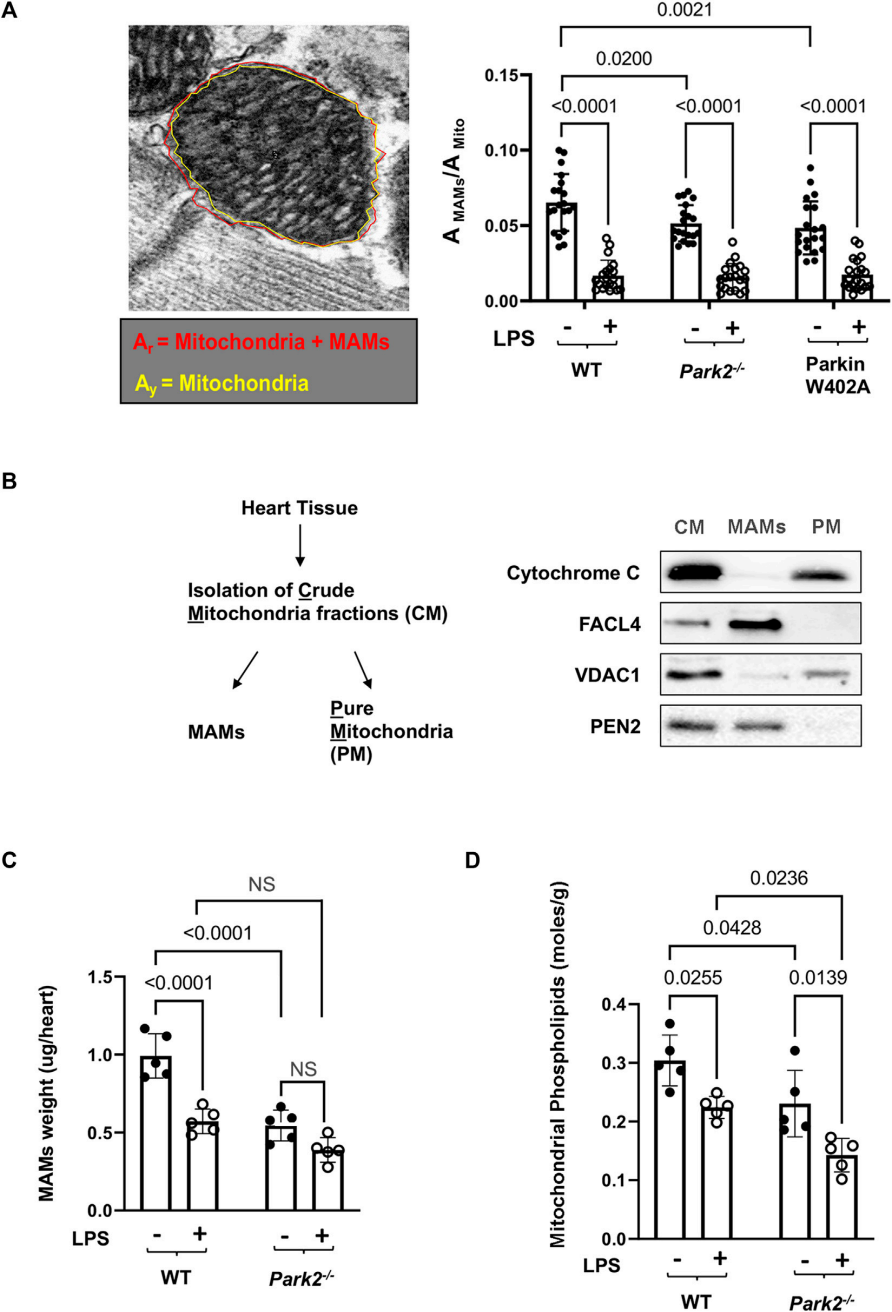
LPS-induced reductions in the total mass and function of cardiac MAMs in WT and *Park2* ^*−/−*^ mice. Mice were given 5 mg/kg LPS or PBS (in shams) *via* i.p., and the heart tissues were harvested 18 h post LPS challenge. **(A).** Quantification of MAM areas was achieved by Image J software based on the images obtained from TEM. The area of mitochondria together with surrounding MAMs, circled in red (A_r_), and the area of mitochondria only, circled in yellow (A_y,_ A_Mito_), were quantified using the polygon selection tool in Image J. The difference between A_r_ and A_y_ is calculated as the area of MAMs, A_MAMs_. More than 20 mitochondria per sample were examined and ratios of A_MAMs_/A_Mito_ were calculated. **(B)**. Validation of the extraction procedure of crude mitochondria (CM), pure mitochondria (PM), and MAMs by ultracentrifugation according to the protocol described in the section of *Methods*. Successful isolation of CM, PM, and MAMs from the heart tissue of WT mice was demonstrated by western blots detecting markers of proteins located in mitochondria, cytochrome C and VDAC1, and proteins reside between mitochondrial outer membrane and ER, FACL4 and PEN2. **(C).** Mass of MAMs was calculated as the total amount of protein in MAM fraction isolated per heart, and the value was normalized by the heart tissue weight. **(D)**. Levels of phospholipids in mitochondrial fractions were measured and results were normalized by the amount of protein. All values are means ± SEM. *p* values with less than 0.05, showing significant differences, are labeled (*n* = 5).

To compare cardiac MAMs of WT with that of the *Park2* ^*−/−*^ counterparts, mice were given 5 mg/kg LPS, and the heart tissue was harvested 18 h post challenge. Since MAMs tightly surround mitochondria, the ultrastructure of MAMs can be examined together with mitochondria under TEM. In the images shown in Figure 3A, red arrows indicate the locations of MAMs. Observation of the TEM images suggested that LPS decreased the amount of MAMs and also caused fragmentation of MAMs, especially in *Park2* ^*−/−*^ mice. To obtain a more precise evaluation, we developed a method to quantify the areas of MAMs and mitochondria based on TEM images using Image J software, as described in Figure 4A. Statistical analysis of the ratios of MAMs to mitochondria clearly showed that LPS triggered a significant decrease in the amount of MAM in the heart tissue of all three mouse strains. In addition, compared with WT, both *Park2* ^*−/−*^ and Parkin W402A showed a decrease in MAMs at baseline.

Further, a biochemical approach was applied to compare the amount and function of MAMs in the heart tissue of WT and *Park2* ^*−/−*^ mice at baseline and following the challenge by LPS. The procedure of isolating MAMs fractions by ultracentrifugation was previously validated (Sun et al., 2021). As illustrated in Figure 4B, successful isolation of MAMs from the total tissue lysates and then subsequential crude mitochondria (CM) fraction was shown by specific markers of subcellular locations. Cytochrome C, an enzyme located in the mitochondrial intermembrane space, was exclusively located in purified mitochondria (PM) but not in MAMs. Mitochondrial outer membrane protein VADC1 was detected primarily in PM. Fatty acid CoA ligase 4 (FACL4), an enzyme enriched in MAMs to facilitate lipid metabolism, and PEN2, a subunit of gamma secretase complex located on the ER membranes, were detected mainly in MAMs but not in PM. As shown in Figure 4C, when the amount of MAM isolation in the heart was quantified based on heart tissue weight, we found that LPS triggered a significant, about a 40% drop in the quantity of MAMs in WT mice. Lacking Parkin expression in *Park2* ^*−/−*^ mice resulted in a near half decrease in the total amount of MAMs at the baseline and about 30% more loss in response to LPS. The result is consistent with the observation of MAM ultrastructure obtained under TEM shown in Figure 3A.

A main function of MAMs is to coordinate the synthesis and transport of phospholipids to other organelles such as mitochondria (Vance, 1990). Since mitochondria are unable to synthesize phospholipids *de novo*, the level of phospholipids inside mitochondria relies entirely on the supply from ER (Voeltz et al., 2002; Vance, 2015; Annunziata et al., 2018). Thus, quantification of mitochondrial phospholipids has been used as an indirect measurement of the transporting function of MAMs (Vance, 1990; Hedskog et al., 2013; Vance, 2015). By this approach, we measured levels of phospholipid accumulation in the mitochondrial fractions from the heart tissue. The result showed that knockout Parkin expression severely reduced phospholipids in mitochondria, suggesting an impaired transporting function of MAMs (Figure 4D).

### W402A Point Mutation in Parkin is not Sufficient to Improve Mitophagy in the Heart During Endotoxemia

**FIGURE 5:**
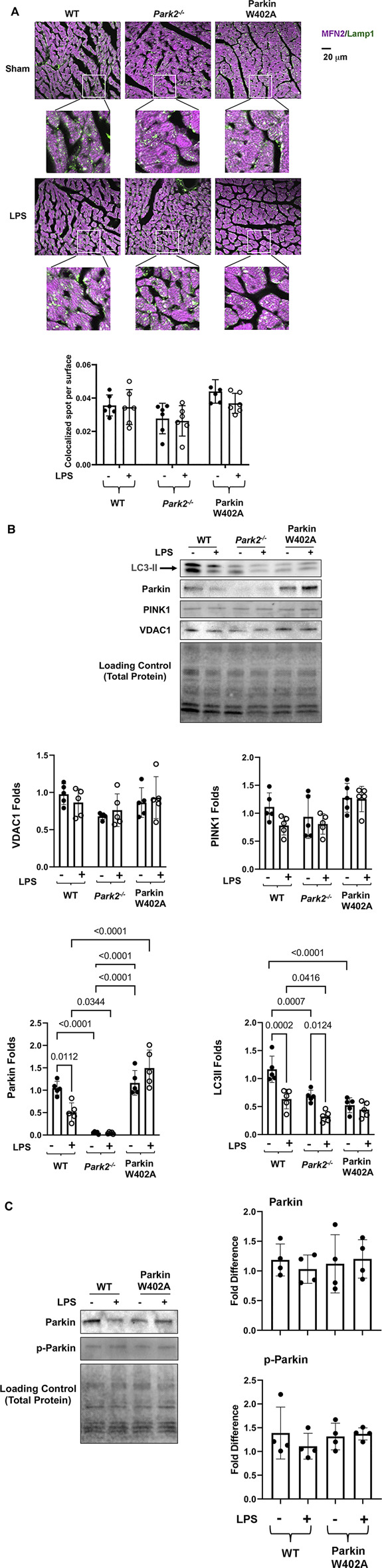
Introducing Parkin W402A mutation is unable to promote cardiac mitophagy during endotoxemia. Mice were given 5 mg/kg LPS or PBS (in shams) *via* i.p., and hearts were harvested 18 h post LPS challenge **(A)**. Heart tissue sections were co-stained with antibodies against lysosomal marker Lamp1 (green) and mitochondrial marker Mfn2 (purple). Colors in white and pale green are resulted from co-localization of the two markers. Each white square circled area was enlarged 9 times for viewing the co-localization status. Additionally, co-localization areas were quantified using Imaris cell imaging software, in which co-localization of Lamp 1 and Mfn2 was determined when the distance between these two signals was ≤0.15 µm. The numbers of co-localization areas were normalized to the cross-sectional area of the Mfn2 fluorescence to permit between-groups statistical comparisons. **(B)** Levels of LC3II, Parkin, PINK1, and VDAC1 in the mitochondrial fractions isolated from the heart tissue were evaluated by western blot. The intensity of signals was quantified by densitometry analysis. **(C)** Levels of Parkin and phosphorylated Parkin (serine 65) were detected in heart tissue lysates by western Blot using antibodies against Parkin and Parkin p-ser65. All values are presented as means ± SEM. *p* values with less than 0.05, showing significant differences, are labeled (*n* = 5).

According to previously established protocol (Sun et al., 2018b), we determined whether introducing Parkin mutation W402A affected cardiac mitophagy in the endotoxemia model by examining mitochondria-lysosome association and the levels of mitophagy/autophagy factors located at mitochondria. As shown in Figure 5A, heart tissue slides were subjected to co-immunostaining with antibodies against mitochondrial protein mitofusin 2 (Mfn2) and lysosomal protein Lamp1. Co-localization of mitochondria and lysosomes, shown in white and pale green, indicate the occurrence of mitophagy. Quantification of Mfn2-Lamp1 co-localization signals showed that there were little LPS-initiated changes in mitochondria-lysosome association in all three mouse strains.

In the mitochondrial fractions isolated from the heart tissue, we examined mitophagy factors Parkin and PINK1, autophagy marker LC3II, and mitochondrial membrane marker protein VDAC by western blot (Figure 5B). Statistical analysis of the signal density showed that LPS caused a significant decrease in mitochondria-located Parkin in the WT but not in Parkin W402A mice. In fact, the level of mitochondria-associated Parkin was significantly higher in LPS-challenged Parkin W402A mice. However, these differences were not associated with any changes in levels of PINK1, suggesting that W402A mutation in Parkin did not promote PINK1 to mitochondria. Further, LPS caused decreases in LC3II in the mitochondria in WT and *Park2* ^*−/−*^ mice, suggesting a reduction in mitophagy by LPS, consistent with our previously published result (Sun et al., 2018b). In Parkin W402A mice, though not changed by LPS, mitochondrial LC3II level was significantly lower than that in the WT at baseline, suggesting mutation W402A in Parkin does not at all improve mitophagy *in vivo.* Since the same amount of mitochondrial protein was loaded on the western blot in this experimental setting, differences in VDAC were neither expected nor observed.

Phosphorylation of Parkin on serine 65 located at its N-terminal ubiquitin-like domain is shown to be essential for Parkin activation (McWilliams et al., 2018). In the heart tissue lysates, Parkin phosphorylation in Parkin W402A mice was compared with that in WT by western Blot using the antibody specific for Parkin serine 65 phosphorylation. The detection revealed no statistically significant difference between these two groups (Figure 5C). Together, these results suggest that W402A mutation in Parkin was not able to promote the Parkin-mediated mitophagy in myocardium under the pathological condition of endotoxemia.

## Discussion

This report summarized our investigation regarding the role of mitophagy factor Parkin in cardiomyopathy induced by endotoxemia. Using a mouse model of endotoxemia, cardiac responses in the mouse strains carrying knockout Parkin expression, *Park2* ^*−/−,*^ and a forced expression of active mutant Parkin W402A were compared with those in the WT counter parts. Data demonstrated that deficiency in Parkin expression exacerbated cardiac dysfunction and cytokine production in response to LPS challenge (Figure 2). Unexpectedly, little improvement of cardiac performance was obtained in Parkin W402A mice under the same condition. Furthermore, evaluation of mitochondrial status, including the ultrastructure, total population, and respiratory function, showed that LPS causes severe impairments in myocardial mitochondria, which response was worsened in *Park2* ^*−/−*^ mice (Figure 3). Since MAM property is essential for maintaining mitochondrial physiology in the heart (Sun et al., 2021), the relationship of Parkin and MAMs was examined in this model. Our results showed that disruption of Parkin expression reduced the total quantity and quantity of cardiac MAMs, either at baseline or under LPS challenge, suggesting that Parkin has a functional role in the regulation of MAMs (Figure 4). Additionally, our evaluation of Parkin W402A mice showed that cardiac mitophagy was not improved by this mutation *in vivo* (Figure 5). Together with published results from other groups, our data suggest that Parkin is essential for supporting proper mitochondrial status *via* Parkin-mediated mitophagy as well as maintaining MAM physiology in the heart under the shock stress of endotoxemia. Mutation W402 on Parkin was not found to associate with detectable benefits of enhancing mitophagy and improving cardiac function.

Research in the past decades has well recognized mitochondrial deficiency as a major signaling to incite functional failure in multiple organs during sepsis. However, the underlying causative mechanisms are still not well-established. MAMs are dynamic interaction domains between mitochondria and ER that sustain mitochondrial health (Patergnani et al., 2011; Vance, 2014), but their pathological role in sepsis is largely unknown. Our recent published investigation detected that endotoxemia caused impairments in myocardial MAMs (Sun et al., 2021). Data summarized in this report suggest that Parkin-dependent regulation of mitochondrial quality control is tightly associated with the properties of MAMs in response to the challenge by endotoxemia (Figure 4). Our data clearly indicate that deficiency in Parkin expression severely impaired cardiac MAMs, shown by disruption in structural integrity, reduction in total mass, and malfunction of transporting phospholipids into mitochondria. This damage in MAMs occurred in parallel with worsened cardiomyopathy induced by endotoxemia, shown by a significant decrease in cardiac contractility, an increase in cytokine production (Figure 2), as well as an intensified adversity of mitochondria status (Figure 3). The newly obtained evidence supports the hypothesis that damage in MAMs is an important cellular component that mediates pathogenesis in mitochondria in septic hearts. Therefore, strategies that protect MAMs may potentially possess therapeutic effectiveness.

The results summarized in this report indicate a Parkin-dependent regulation of cardiac MAMs, suggesting that the formation of MAMs may demand an active machinery of Parkin/PINK1 mitophagy. In this regard, Parkin and its partner protein PINK1 were previously found to be located at MAMs, suggesting a plausible involvement of Parkin in regulating MAM properties (Michiorri et al., 2010; Choubey et al., 2014; Gelmetti et al., 2017). On the other hand, *in vitro* studies have linked the initiation of PINK1/Parkin mitophagy to a signal of phosphorylation on Mfn2 (Shiba-Fukushima et al., 2012; Chen and Dorn, 2013; Jin and Youle, 2013), which is by far the most important identified key regulatory factor of MAMs (Cipolat et al., 2004; de Brito and Scorrano, 2008). Additionally, MAMs are also known as a signaling hub harboring key molecules during protein sorting, ER stress, apoptosis, inflammation, and autophagy (Poston et al., 2013; van Vliet et al., 2014). Therefore, a proper maintenance of MAM status is likely a necessary component for starting mitophagy as well as regulating other cellular functions that depend on energy supply from the mitochondria. Though the potential signaling interactions between Parkin and molecules in MAMs is beyond the scope of this report, our ongoing investigation is set up to apply omics approaches to obtain a comprehensive signaling diagram in near future.

The upstream signaling regulation of MAMs may involve autophagy. Previously, we reported that insufficient, maladaptive autophagy occurs during cardiomyopathy in endotoxemia, and promoting autophagy *via* Beclin-1 attenuates mitochondrial damage and improves cardiac performance at the same condition (Sun et al., 2018a). Data also suggested that this Beclin-1-mediated protection on mitochondria is accomplished *via* a selective activation of Parkin/Pink1 mitophagy (Sun et al., 2018a), which target mitochondria with lost membrane potential and subsequently bring these dysfunctional mitochondria to autophagosomes for degradation (Narendra et al., 2008; Narendra et al., 2010). Our recent study further revealed that targeted activation of Beclin-1, either genetically or pharmacologically, is capable of protecting the structure and function of cardiac MAMs from endotoxemia challenge (Sun et al., 2021). Together, these investigations suggest that sepsis-induced myocardial mitochondrial damage is a result of MAM abnormality stimulated by inadequate autophagic responses.

Research from other and ours support the notion that PINK1/Parkin mitophagy is cardiac-protective, an adaptive response, during sepsis as well as other pathological conditions of heart failure (Sun et al., 2018b) (Kubli et al., 2013; Piquereau et al., 2013). A structure-guided mutagenesis screen and *in vitro* cell culture studies identified point mutation W403A in Parkin with enhanced Parkin activity and increased PINK1-Parkin mitophagy (Tang et al., 2017; Yi et al., 2019). We therefore designed *in vivo* experiments to evaluate the cardiac responses in mice carrying knock-in expression of Parkin W402A (human W403A). However, when challenged with LPS, introducing this mutant Parkin neither improved cardiac function nor protected myocardial mitochondria (Figures 2, 3). It is also worthy to point out that our analysis using TEM did not detect any sign of increase in mitophagy, in which situation mitochondria would be engulfed by the double-membrane structure of autophagosomes and/or autolysosomes. Our further analysis of mitophagy and Parkin phosphorylation at serine 65, which represents its activation, did not detect any difference between the WT and Parkin W402A mice (Figure 5), confirming little impact provided by W402A on mitophagy or activation of Parkin. It remains possible that this mutation in Parkin may convey Parkin function in a tissue or cell type specific feature. Alternatively, the level of Parkin activation may need to be optimized to accomplish proper mitophagy. Nonetheless, current data from our investigation suggest that point mutation W402A (human W403A) in Parkin by itself is not sufficient to provide cardiac protection or promote mitophagy *in vivo* under the challenge by endotoxemia. Thus, the results suggest that point mutation W402 (human W403) in Parkin is unable to promote Parkin activation *in vivo.*

## Data Availability

The raw data supporting the conclusions of this article will be made available by the authors, without undue reservation.
